# Critical assessment of variant prioritization methods for rare disease diagnosis within the rare genomes project

**DOI:** 10.1186/s40246-024-00604-w

**Published:** 2024-04-29

**Authors:** Sarah L. Stenton, Melanie C. O’Leary, Gabrielle Lemire, Grace E. VanNoy, Stephanie DiTroia, Vijay S. Ganesh, Emily Groopman, Emily O’Heir, Brian Mangilog, Ikeoluwa Osei-Owusu, Lynn S. Pais, Jillian Serrano, Moriel Singer-Berk, Ben Weisburd, Michael W. Wilson, Christina Austin-Tse, Marwa Abdelhakim, Azza Althagafi, Giulia Babbi, Riccardo Bellazzi, Samuele Bovo, Maria Giulia Carta, Rita Casadio, Pieter-Jan Coenen, Federica De Paoli, Matteo Floris, Manavalan Gajapathy, Robert Hoehndorf, Julius O. B. Jacobsen, Thomas Joseph, Akash Kamandula, Panagiotis Katsonis, Cyrielle Kint, Olivier Lichtarge, Ivan Limongelli, Yulan Lu, Paolo Magni, Tarun Karthik Kumar Mamidi, Pier Luigi Martelli, Marta Mulargia, Giovanna Nicora, Keith Nykamp, Vikas Pejaver, Yisu Peng, Thi Hong Cam Pham, Maurizio S. Podda, Aditya Rao, Ettore Rizzo, Vangala G. Saipradeep, Castrense Savojardo, Peter Schols, Yang Shen, Naveen Sivadasan, Damian Smedley, Dorian Soru, Rajgopal Srinivasan, Yuanfei Sun, Uma Sunderam, Wuwei Tan, Naina Tiwari, Xiao Wang, Yaqiong Wang, Amanda Williams, Elizabeth A. Worthey, Rujie Yin, Yuning You, Daniel Zeiberg, Susanna Zucca, Constantina Bakolitsa, Steven E. Brenner, Stephanie M. Fullerton, Predrag Radivojac, Heidi L. Rehm, Anne O’Donnell-Luria

**Affiliations:** 1 Division of Genetics and Genomics, Boston Children’s Hospital, Harvard Medical School, Boston, MA USA; 2 Program in Medical and Population Genetics, Broad Institute of MIT and Harvard, Cambridge, MA USA; 3 Center for Genomic Medicine, Massachusetts General Hospital, Boston, MA USA; 4 Department of Neurology, Brigham and Women’s Hospital, Harvard Medical School, Boston, MA USA; 5 Computer, Electrical and Mathematical Sciences and Engineering Division (CEMSE), King Abdullah University of Science and Technology (KAUST), 23955-6900 Thuwal, Saudi Arabia; 6 Computational Bioscience Research Center (CBRC), King Abdullah University of Science and Technology (KAUST), 23955-6900 Thuwal, Saudi Arabia; 7 Computer Science Department, College of Computers and Information Technology, Taif University, Taif, Saudi Arabia; 8 Biocomputing Group, Department of Pharmacy and Biotechnology, University of Bologna, Bologna, Italy; 9 enGenome Srl, Pavia, Italy; 10 Department of Electrical, Computer and Biomedical Engineering, University of Pavia, Pavia, Italy; 11 Department of Agricultural and Food Sciences, University of Bologna, Bologna, Italy; 12 Invitae, San Francisco, CA USA; 13 Codon One, Louvain, EU Belgium; 14 Department of Biomedical Sciences, University of Sassari, Sassari, Italy; 15 Center for Computational Genomics and Data Science, The University of Alabama at Birmingham, Birmingham, AL USA; 16 Department of Genetics, Heersink School of Medicine, The University of Alabama at Birmingham, Birmingham, AL USA; 17 Hugh Kaul Precision Medicine Institute, The University of Alabama at Birmingham, Birmingham, AL USA; 18 William Harvey Research Institute, Barts & The London School of Medicine and Dentistry, Queen Mary University of London, Charterhouse Square, London, UK; 19 TCS Research, Tata Consultancy Services (TCS) Ltd, Deccan Park, Madhapur, Hyderabad India; 20 Khoury College of Computer Sciences, Northeastern University, Boston, MA USA; 21 Department of Molecular and Human Genetics, Baylor College of Medicine, Houston, TX USA; 22 Structural and Computational Biology and Molecular Biophysics Program, Baylor College of Medicine, Houston, TX USA; 23 Computational and Integrative Biomedical Research Center, Baylor College of Medicine, Houston, TX USA; 24 Center for Molecular Medicine, Pediatric Research Institute, Children’s Hospital of Fudan University, Shanghai, China; 25 Institute for Genomic Health, Icahn School of Medicine at Mount Sinai, New York, NY USA; 26 Department of Genetics and Genomic Sciences, Icahn School of Medicine at Mount Sinai, New York, NY USA; 27 University of Medicine and Pharmacy, Hue University, Hue, Vietnam; 28 Institute of Clinical Physiology (IFC), CNR, Via Moruzzi 1, 56124 Pisa, Italy; 29 University of Siena, Siena, Italy; 30 Department of Electrical and Computer Engineering, Texas A&M University, College Station, TX USA; 31 Department of Computer Science and Engineering, Texas A&M University, College Station, TX USA; 32 Institute of Biosciences and Technology and Department of Translational Medical Sciences, College of Medicine, Texas A&M University, Houston, TX USA; 33 Independent Consultant, Ovodda, Italy; 34 Department of Plant and Microbial Biology and Center for Computational Biology, University of California, Berkeley, CA USA; 35 Department of Bioethics and Humanities, University of Washington School of Medicine, Seattle, WA USA; 36 CTGLab, Institute of Informatics and Telematics (IIT), CNR, ViaMoruzzi 1, 56124 Pisa, Italy

**Keywords:** Rare disease, Genome sequencing, Genome interpretation, Variant prioritization, Best practices

## Abstract

**Background:**

A major obstacle faced by families with rare diseases is obtaining a genetic diagnosis. The average "diagnostic odyssey" lasts over five years and causal variants are identified in under 50%, even when capturing variants genome-wide. To aid in the interpretation and prioritization of the vast number of variants detected, computational methods are proliferating. Knowing which tools are most effective remains unclear. To evaluate the performance of computational methods, and to encourage innovation in method development, we designed a Critical Assessment of Genome Interpretation (CAGI) community challenge to place variant prioritization models head-to-head in a real-life clinical diagnostic setting.

**Methods:**

We utilized genome sequencing (GS) data from families sequenced in the Rare Genomes Project (RGP), a direct-to-participant research study on the utility of GS for rare disease diagnosis and gene discovery. Challenge predictors were provided with a dataset of variant calls and phenotype terms from 175 RGP individuals (65 families), including 35 solved training set families with causal variants specified, and 30 unlabeled test set families (14 solved, 16 unsolved). We tasked teams to identify causal variants in as many families as possible. Predictors submitted variant predictions with estimated probability of causal relationship (EPCR) values. Model performance was determined by two metrics, a weighted score based on the rank position of causal variants, and the maximum F-measure, based on precision and recall of causal variants across all EPCR values.

**Results:**

Sixteen teams submitted predictions from 52 models, some with manual review incorporated. Top performers recalled causal variants in up to 13 of 14 solved families within the top 5 ranked variants. Newly discovered diagnostic variants were returned to two previously unsolved families following confirmatory RNA sequencing, and two novel disease gene candidates were entered into Matchmaker Exchange. In one example, RNA sequencing demonstrated aberrant splicing due to a deep intronic indel in *ASNS*, identified in *trans* with a frameshift variant in an unsolved proband with phenotypes consistent with asparagine synthetase deficiency.

**Conclusions:**

Model methodology and performance was highly variable. Models weighing call quality, allele frequency, predicted deleteriousness, segregation, and phenotype were effective in identifying causal variants, and models open to phenotype expansion and non-coding variants were able to capture more difficult diagnoses and discover new diagnoses. Overall, computational models can significantly aid variant prioritization. For use in diagnostics, detailed review and conservative assessment of prioritized variants against established criteria is needed.

**Supplementary Information:**

The online version contains supplementary material available at 10.1186/s40246-024-00604-w.

## Introduction

Genome sequencing (GS) is increasingly becoming a standard genetic test for rare disease diagnosis and research [1, 2], capturing variants in both the coding and non-coding genomic space, and resulting in approximately 75,000 rare variants at ≤ 1% population allele frequency, per individual, for clinical consideration [3]. The reported diagnostic gap, where > 50% of rare disease patients remain undiagnosed, therefore becomes more of a question of our capability to prioritize and interpret clinical relevance, rather than to capture variants [4, 5]. The current standards for determining variant pathogenicity are defined by the American College of Medical Genetics and Genomics and the Association for Molecular Pathology (ACMG/AMP) and refined by ClinGen [6–8], and require in-depth curation of variants to reach pathogenic (P) or likely pathogenic (LP) designation. A well-recognized analytical obstacle to diagnosis, is the need to prioritize a manageable number of variants for clinical review, requiring integration of evidence such as population allele frequency and in silico prediction of deleteriousness, in the context of phenotype and segregation of the variant(s) in the family [9].

To help bridge knowledge gaps in variant interpretation, a broad-spectrum of in silico prediction tools of variant impact have been developed [10, 11] and large population databases have been generated to provide allele frequencies [3, 12, 13], both enabling the detection of rare variants and enabling assignment of metrics such as loss-of-function and missense constraint genome-wide [3]. The precise nature by which these tools are most effectively integrated in the context of phenotype and segregation to pinpoint genetic diagnoses in rare disease families remains an open question. This has spurred the development of numerous computational algorithms integrating machine learning, artificial intelligence, natural language processing, and Human Phenotype Ontology (HPO) semantic similarity, among others [9]. Each variant prioritization method reports the ability to detect clinically relevant variants from sequencing data; however, independent assessments on unpublished datasets are often not performed at all nor by a variety of developers or users. We therefore developed a challenge within the Critical Assessment of Genome Interpretation (CAGI) framework [14] with the goal to evaluate computational methods independently and objectively in a real-life diagnostic setting. We utilized data from the Rare Genomes Project (RGP) (raregenomes.org/), a study generating and analyzing research GS data from a diverse range of families seeking a molecular diagnosis for a rare disease. The aim of the RGP study is to identify variants of clear or potential diagnostic relevance for clinical validation and to return these variants to the families via their local physicians. For the CAGI6-RGP challenge, predictors were provided with variants from GS and phenotype data standardized as HPO terms [15] from a subset of solved and unsolved RGP families, and were tasked with identifying the causal variant(s) in as many families, and at the highest rank, as possible.

Here, we report on the format, assessment, and outcome of the challenge, including lessons learnt from exploration of differences in performance across prediction strategies and provision of method reports from participating teams.

## Methods

### Sequencing, variant calling, and analysis by the RGP team

Genomic data were obtained by sequencing DNA purified from blood. Sequencing was performed by the Broad Institute Genomics Platform on an Illumina sequencer to 30 × depth on average. Raw sequence reads were mapped to the GRCh38 reference genome with GATK version 4.1.8.0 [16] and variants were subsequently called in the form of single nucleotide variants (SNVs) and small insertions/deletions (indels). All data were analyzed by expert RGP variant analysts using a series of predefined searches in *seqr*, an open-source, web-based genomic analysis tool for family-based monogenic disease analysis (seqr.broadinstitute.org/). This encompasses “De Novo/Dominant” and “Recessive” searches with both “Restrictive” and “Permissive” thresholds for reports of pathogenicity, annotations of functional consequence and predicted deleteriousness, allele frequency, and call quality, described in detail here [17]. Our analysts assess all variants returned by these searches in the context of data from external resources linked in *seqr*, including gene-level data (OMIM, PubMed, DECIPHER) [18–20], transcript-level data (Genotype-tissue Expression [GTEx]) [21], and functional data, such as mouse models [22, 23]. Structural variants (SVs) were not included in this challenge, but have been analyzed and found to be non-contributory by the RGP team independent from the CAGI challenge.

### Challenge datasets

Two datasets were provided for the CAGI6-RGP challenge, a training set and a test set. For each, a joint variant call format (VCF) file was provided to the CAGI6 organizers for use in the challenge. In addition to the genomic data, clinical phenotype descriptions from patient-provided information and review of medical records by a genetic counselor or medical geneticist were provided in HPO nomenclature. The diversity of phenotypes represented the range of clinical presentations routinely seen in patients referred for genetic testing. The family structure and affected status of each sequenced individual were provided, identifying the proband, sibling, mother, and father, as applicable.

For training and contextual purposes, GS and HPO data from 35 solved RGP families were provided along with the causal variant(s) identified by the RGP team. Ancestry was not provided but was imputed for the probands using the principal component analysis and random forest model used for the Genome Aggregation Database (gnomAD) [3]. Overall, the training set consisted of six proband-only families, three duos (proband and one biological parent), and 26 trios (proband and both biological parents). The inheritance mode of the diagnoses spanned de novo (n = 21), recessive (n = 8), X-linked recessive (n = 1), or unconfirmed (n = 5). Most responsible variants had been reported in the ClinVar database as P and/or LP [24] at the time the challenge was announced (May 3, 2021) (Additional file 2: Table S1).

For test purposes, the RGP team selected 30 families for inclusion in the challenge. Fourteen were solved and 16 were unsolved after standard analysis. The solved families in the test set were selected more stringently than for the training set, according to the following criteria: (i) the responsible gene has an established Mendelian disease-association as per the Online Mendelian Inheritance in Man database (OMIM) [19] and/or published literature at the time the challenge was announced, (ii) the responsible variant(s) must not have been reported as P/LP in the ClinVar database or listed in/reported as a disease mutation (DM) in the HGMD Professional database [25] at the time the challenge was announced (May 3, 2021), and (iii) the variant(s) were classified as P, LP, or variant of uncertain significance (VUS) with evidence that is close to LP according to the ACMG/AMP guidelines [6]. The causal variants in all 14 solved families had been discussed by the RGP multi-disciplinary team of physicians, genetic counselors, analysts, and molecular geneticists, and had been returned to the family via a local clinician following confirmation in a CLIA certified laboratory. The local clinicians concurred that the variants were diagnostic. The submission of these variants from RGP participants to ClinVar was intentionally delayed for the duration of the challenge. Additional file 2: Table S2 displays the answer key for the 30 families in the test set. Overall, the test set consisted of two proband-only families, three duos, 23 trios (proband and both biological parents), and two quads (proband, affected biological sibling, and both biological parents). From the larger RGP cohort, we selected 16 unsolved families with high likelihood to be Mendelian (scored 4 or 5 by scoring [1–5] for likelihood of there being a Mendelian cause for the phenotype independently by two clinical geneticists), prioritizing trios (15 trios, one quad) and aiming for a number of families comparable to the number of solved families.

**Fig. 1:**
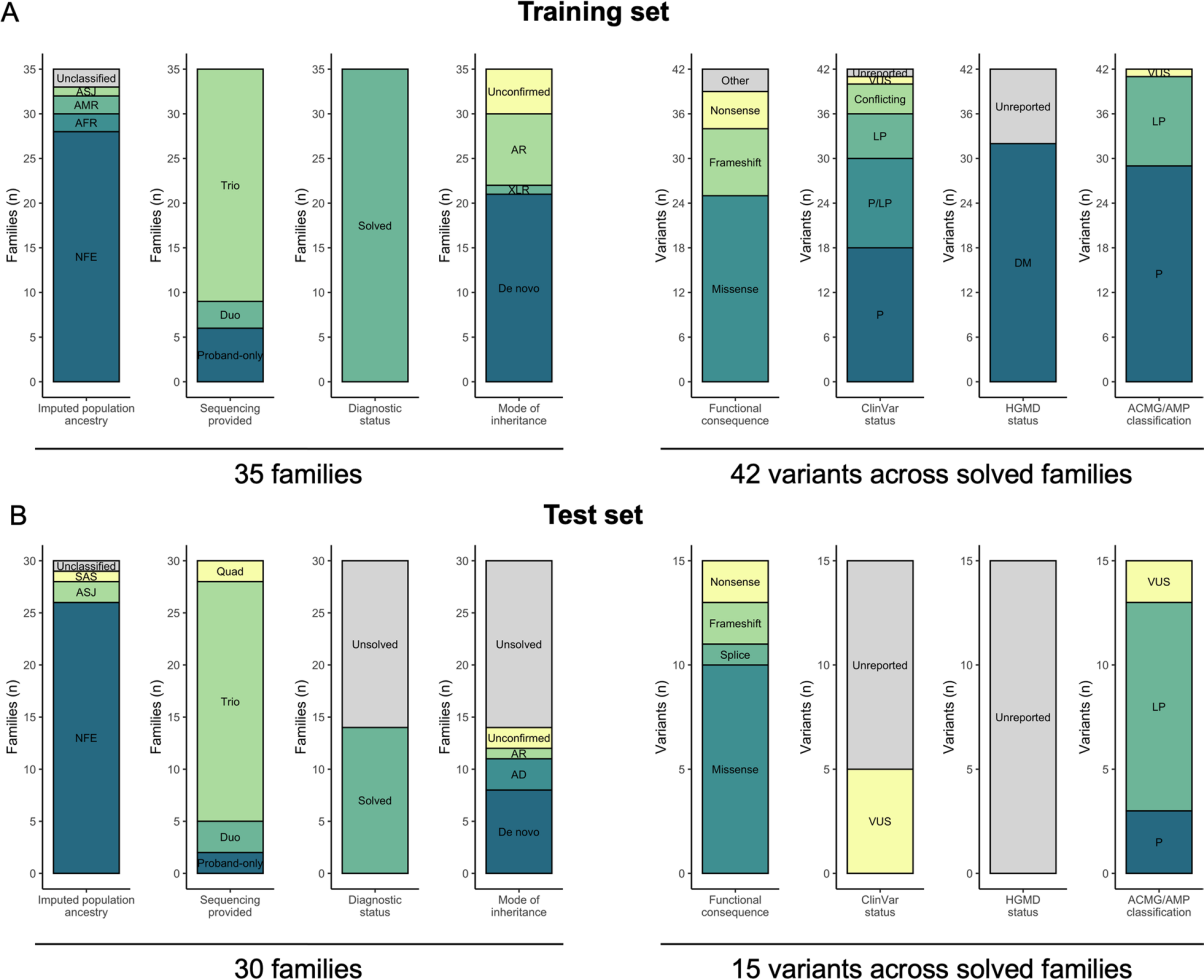
CAGI6-RGP challenge overview of selected families. Summary of the 35 training set families (all solved) and 30 test set families (14 solved, 16 unsolved). Imputed population ancestry, the amount of familial sequencing data provided (proband-only, duo, trio, or quad), diagnostic status, and mode of inheritance of the causal variant(s) is displayed by family. For all returnable diagnostic variants in the solved families in each set, the functional consequence according to the Variant Effect Predictor (VEP), ClinVar and HGMD reporting status at the time of announcement of the challenge (May 3, 2021), and ACMG/AMP classification are displayed by variant. NFE, Non-Finnish European; AFR, African/African American; AMR, Admixed American; ASJ, Ashkenazi Jewish; SAS, South Asian; AD, autosomal dominant; XLR, X-linked recessive; AR, autosomal recessive; P, pathogenic; LP, likely pathogenic; VUS, variant of uncertain significance; DM, disease mutation

A summary of the core features of the families and diagnostic variants in the CAGI-RGP challenge training and test sets is depicted in Fig. 1.

### Challenge format

As part of CAGI6, the RGP-CAGI challenge was publicly announced on the CAGI website (genomeinterpretation.org/challenges.html) on May 3, 2021, and open for submissions on June 8, 2021. Teams were eligible to participate upon creating a CAGI account and a Synapse account, and signing an agreement to adhere to the CAGI Data Use Agreement and Anonymity Policy. The submission deadline was October 11, 2021. Participating teams were tasked to provide a genetic diagnosis to as many probands from the 30 families in the test set as possible by submitting predictions for each proband’s causal variant(s). The 14 solved families were included in the challenge to evaluate the performance of each model in prioritizing the causal variants (true positives). The unsolved families were included with the goal of the identifying novel, potentially causal, variants for further clinical and experimental assessment followed where possible by return to the families. The number of solved and unsolved families was not disclosed in the challenge description to allow the participating teams to perform the task in a manner that reflects analysis in the clinical setting. Teams were able to submit up to 100 variant predictions per proband, ranked by causal likelihood, from a maximum of six different models. The submission format, a tab-delimited text file, accepted both single (one variant per line) and proposed compound heterozygous (two variants per line) predictions. For each variant, teams were required to provide an estimated probability of causal relationship (EPCR) value for the variants being causal on a scale of 0 to 1, with 1 indicating highest certainty. An example submission file and a validation script were provided. Predictors were informed that assessors will review how often the true positive causal variants were the top variant(s) returned (e.g., in the top 5, 10, 20, 50, or 100 variants) but were not informed of the details of the assessment metrics. Teams were required to delete the raw and any derived RGP data after the conclusion of the challenge.

### Assessment of model performance across solved families

Formatting errors in the submission files were corrected, and redundant, duplicate, and incomplete submissions were removed. Causal variant predictions for each solved proband were assessed by an independent assessor (author S.L.S). The assessor was blinded to the identity and methods of the participating teams throughout assessment. The identities of the participating teams were only revealed once the analysis was completed. The following two numeric metrics were considered:

(i) **Mean rank points:** The mean of a weighted point allocation system based on the rank position of the true positive causal variant(s) in the solved probands within the top five (100 points), top 10 (50 points), top 20 (25 points), top 50 (10 points), or top 100 (5 points) variant predictions per proband. Model performance was subsequently ranked by the mean points awarded per proband.
(ii) **F-max:** The F-measure, a harmonic mean between the precision and recall for causal variant prediction in the solved probands, was calculated for all unique EPCR values for each model. The maximum F-measure (F-max) [26], corresponding EPCR threshold, and mean number of predictions submitted per proband at and above this EPCR threshold were defined for each model and model performance was ranked by the resultant F-max value.

For both numeric metrics, a bootstrapped standard error (SE) [27] was calculated over 1,000 bootstrapped samples from the probands of the 14 solved families in the test set only.

The causal variants in the answer key had been formally classified as P, LP, or VUS leaning towards LP according to the ACMG/AMP guidelines; however, for the purpose of matching the teams’ predictions to the answer key, all variants were treated equivalently. In the case that a correct causal variant was submitted in combination with a second non-causal variant in a proposed biallelic, recessive prediction, the prediction was considered incorrect. For P27, a proband from a family where both the proband and the affected sibling had inherited two paternal variants in *cis* (6 base pairs apart), where it is unknown if both or only one of the variants is required and both variants were considered equally likely to be causal by the RGP team (Additional file 2: Table S2), the highest-ranked variant prediction for either one of the two variants by the respective model was retained and the other was removed from the analysis.

### Assessment of novel putative causal variants across solved and unsolved families

Following assessment of model performance, predictions from top performing models that (i) deviated from the answer key in the solved probands and (ii) were submitted for the unsolved probands, were critically evaluated in the rare disease genomics web-based analysis tool *seqr* [17]. Putative causal variants were discussed by the RGP team and, where possible, were pursued by: (i) functional validation by RNA sequencing, (ii) SV analysis in a separate call set generated by the GATK-SV pipeline [28] and manually reviewed in the Integrative Genome Viewer (IGV) [29] to search for a compound heterozygous variant in the case of recessive disease genes, and (iii) submission to the Matchmaker Exchange (matchmakerexchange.org/) via *seqr* in the case of candidate novel disease-genes.

### Ethical considerations

The challenge data were derived from patients with rare, suspected monogenic conditions and their close biological relatives, and included families who are medically underserved [30]. Identification of putative causal variants, i.e., causal with respect to the clinical phenotype under investigation, may, if confirmed, be important for tailoring clinical interventions and obtaining social services. We did not actively search for variants unrelated to the rare condition in the family but the consent allows us to optionally provide clinical confirmation of secondary findings if they are incidentally discovered. For the purpose of this challenge, participating teams were told that pathogenic variants unrelated to the proband’s phenotype, such as might be identified as secondary or incidental variants in this challenge [31], should not be returned. All RGP participants have a consent video or phone call with a trained research coordinator to review the study protocol which includes provisions for sharing de-identified data and provide signed informed consent (Mass General Brigham IRB protocol 2016P001422). An institutionally signed (Broad-Northeastern) data transfer agreement was executed. We applied a registered access model [32] where all CAGI6 challenge predictors were required to sign and adhere to the CAGI Data Use Agreement (genomeinterpretation.org/data-use-agreement.html) but institutional signatures were not required.

## Results

### Summary of submissions

Sixteen teams participated in the challenge, submitting predictions from a total of 52 models (median three models per team, range 1–6). Five teams elected to remain anonymous in the reporting, including one team (Team 6) that discovered a bug in their code during assessment and subsequently withdrew from the challenge. Between 0 and 100 variant predictions (single or proposed biallelic) were submitted per proband (range 0–100, median 100, mean 65). EPCR values ranged from 0–1 (median 0.32, mean 0.38) (Additional file 1: Fig. S1). Ninety percent of predictions were single variants and 10% were possible compound heterozygous variants. Over half (53%) of all variant predictions were in established disease-associated genes according to OMIM. Eighty-four percent of predictions were in the coding sequence or direct splice region, as defined by the Ensembl VEP (i.e., within 1–3 bases of the exon, 3–8 bases of the intron, or in the splice polypyrimidine tract). Concordance between models for the top five ranked predictions per proband across all 30 families in the test set ranged from 0–1 (mean 0.09, standard deviation [SD] 0.15) and was only significant between different models from the same team, not between different models from different teams (Additional file 1: Fig. S2).

### Summary of numeric assessment of model performance and methodology

**Fig. 2:**
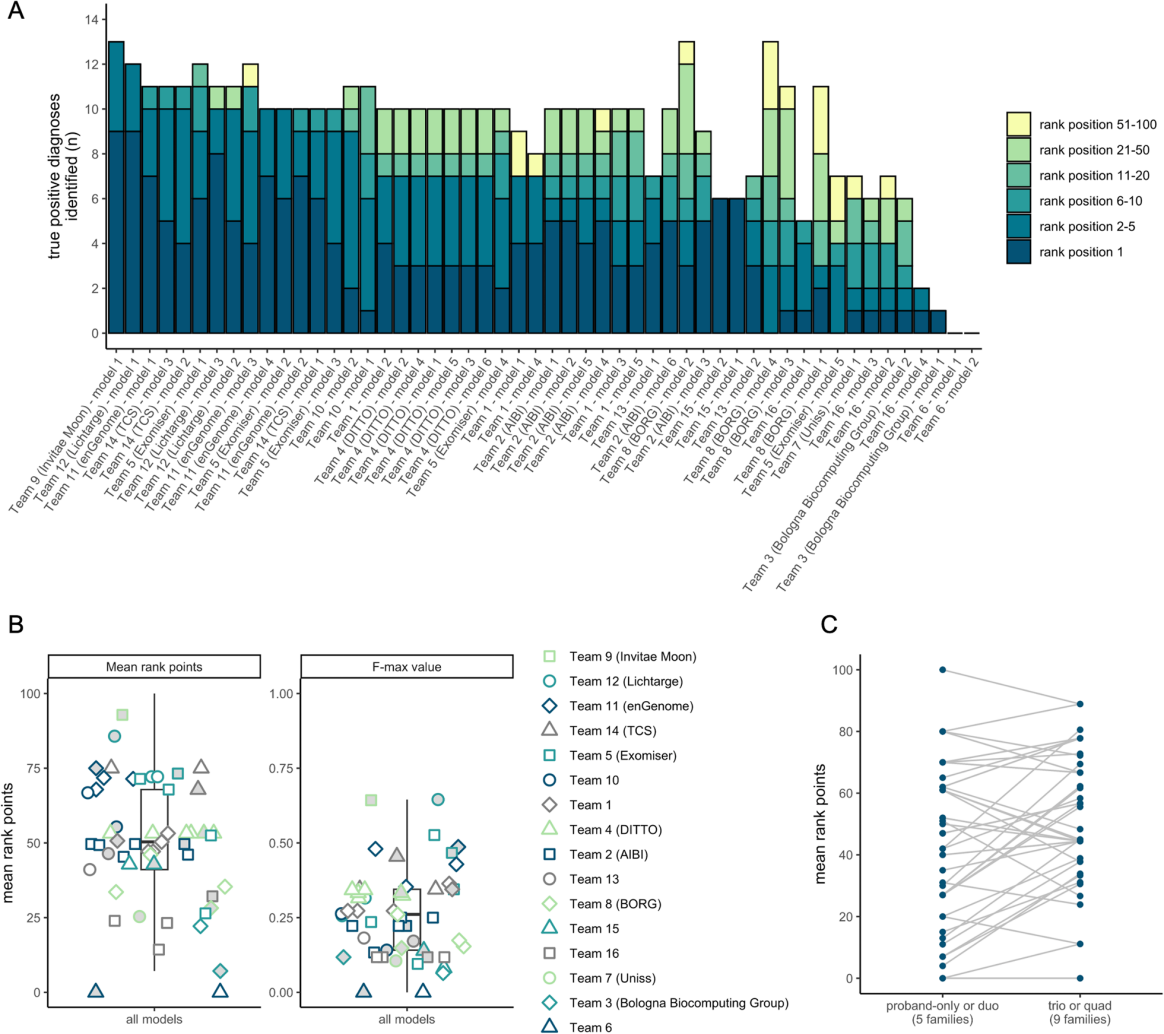
Results of assessment using the 14 solved families (true positives). **A** Number of true positive diagnoses (y-axis) identified per model (x-axis) colored by the rank position of the causal variants in the 14 solved probands. Models are ordered by their performance according to the mean rank points metric (Table 2). Team names are provided except for teams that elected to remain anonymous. **B** Results of the mean rank points and F-max value numeric assessment metrics by team and model. Model 1, the primary model, for each team is indicated by the grey fill. **C**, Performance of models, according to the mean rank points awarded, comparing families with proband-only or duo data (i.e., an incomplete trio/quad) versus trio or quad data (i.e., a complete trio/quad)

Overall, model performance was highly variable (Fig. 2A). All causal variants in the answer key were predicted within the first five rank positions by at least one model (Table 1). Our selected numeric assessment metrics for each submitted model are displayed in Table 2 and are depicted in Fig. 2B.

**Table 1:** Detection of causal variants

Proband	Gene symbol	Sequencing provided	Inheritance	Known gene for phenotype	Variant call quality	Variant allele frequency (gnomAD v2 exomes, v3 genomes)	Variant functional consequence	Variant deleteriousness prediction	ACMG/AMP classification	*seqr* search returning the variant (total variants returned by search)	Number of models with the causal variant(s) at rank 1	Number of models with the causal variant(s) at rank 1–5	Number of models with the causal variant(s) at rank 1–10	Number of models with the causal variant(s) at rank 1–20	Number of models with the causal variant(s) at rank 1–50	Number of models with the causal variant(s) at rank 1–100
P28	*FGFR2*	Trio, unaffected parents	De novo	Yes	DP 27, GQ 99, AB 0.48	Absent	Missense	REVEL 0.98	P	De Novo/Dominant Restrictive (3)	36	**46**	46	47	47	47
P11	*TPM2*	Proband-only	Dominant	Yes	DP 48, GQ 99, AB 0.54	Absent	Missense	REVEL 0.89	VUS	De Novo/Dominant Restrictive (191)	17	**40**	41	44	47	47
P4	*NALCN*	Trio, unaffected parents	De novo	Yes	DP 34, GQ 99, AB 0.44	Absent	Missense	REVEL 0.94	LP	De Novo/Dominant Restrictive (3)	27	**37**	39	40	44	46
P7	*EHMT1*	Trio, unaffected parents	De novo	Yes	DP 50, GQ 99, AB 0.36	Absent	Frameshift	pLoF	LP	De Novo/Dominant Permissive (23)	27	**34**	36	37	38	40
P2	*FAM111B*	Proband-only	Dominant	Yes	DP 31, GQ 99, AB 0.55	Absent	Missense	REVEL 0.55	LP	De Novo/Dominant Restrictive (202)	15	**27**	33	35	41	41
P21	*BICC1*	Trio, unaffected parents	De novo	Yes	DP 31, GQ 99, AB 0.29	Absent	Missense	REVEL 0.19	LP	De Novo/Dominant Restrictive (2)	3	**21**	25	28	33	36
P24	*KMT2D*	Trio, unaffected parents	De novo	Yes	DP 49, GQ 99, AB 0.33	Absent	Missense	REVEL 0.36	LP	De Novo/Dominant Restrictive (3)	9	**19**	28	30	34	34
P22	*GRIN2A*	Duo, unaffected mother	Unknown	Yes	DP 25, GQ 99, AB 0.44	Absent	Nonsense	pLoF	P	De Novo/Dominant Restrictive (96)	6	**19**	23	32	34	34
P23	*GNAI1*	Trio, unaffected parents	De novo	Yes	DP 42, GQ 99, AB 0.45	Absent	Missense	REVEL 0.82	LP	De Novo/Dominant Restrictive (3)	14	**19**	19	21	29	31
P16	*DLG4*	Trio, unaffected parents	De novo	Yes	DP 27, GQ 99, AB 0.44	Absent	Frameshift	pLoF	LP	De Novo/Dominant Restrictive (4)	10	**18**	22	24	25	26
P27	*TUBB8*	Quad, affected sibling	Dominant	Yes	DP 26, GQ 99, AB 0.46	Absent	Missense	REVEL 0.31	VUS	Custom Panel Restrictive (4)*	11	**18**	20	22	23	23
P19	*CLTC*	Trio, unaffected parents	De novo	Yes	DP 39, GQ 99, AB 0.41	Absent	Splice acceptor	pLoF	LP	De Novo/Dominant Restrictive (4)	8	**13**	16	17	18	18
P5	*PI4KA*	Duo, unaffected father	Recessive	Phenotype expansion	DP 36, GQ 99, AB 0.56; DP 45, GQ 99, AB 0.56	0.00007230; Absent	Nonsense; Missense	pLoF; REVEL 0.73	P; LP	Recessive Restrictive (12)	6	**9**	12	12	15	18
P6	*KCND2*	Duo, unaffected father	Unknown	Yes	DP 37, GQ 99, AB 0.57	Absent	Missense	REVEL 0.84	LP	De Novo/Dominant Restrictive (107)	0	**3**	5	12	22	26

The 14 solved (true positive) cases are displayed with the causal gene, inheritance pattern, functional consequence of the causal variant(s), amount of familial sequencing provided in the challenge, and the ACMG/AMP classification of the variant(s). The number of models, out of 52, ranking the variant at position 1, 1–5, 1–10, 1–20, 1–50, and 1–100 are depicted. The probands are displayed in decreasing order by the number of causal variants submitted at rank position 1–5 by the models (emboldened), considered reasonable performance for a prediction metric. *Infertility gene panel search (93 genes) does not consider segregation. P, pathogenic; LP, likely pathogenic; VUS, variant of uncertain significance; pLoF, predicted loss-of-function (frameshift, nonsense, splice acceptor, and splice donor variants)

**Table 2:** Numeric assessment metrics for all models

Model #	Team	Causal variant(s) at rank 1	Causal variant(s) at rank 1–5	Causal variant(s) at rank 1–10	Causal variant(s) at rank 1–20	Causal variant(s) at rank 1–50	Causal variant(s) at rank 1–100	Mean rank points ± SE	F-max value ± SE	F-max EPCR threshold	Mean predictions per proband above F-max EPCR threshold ± SD	Performance by mean rank points	Performance by F-max value
1	Team 9 (Invitae Moon)	9	13	13	13	13	13	92.9 ± 7.3	0.64 ± 0.12	0.88	1 ± 0	1	2
1	Team 12 (Lichtarge)	9	12	12	12	12	12	85.7 ± 9.3	0.65 ± 0.11	0.6	1.21 ± 0.43	2	1
1	Team 11 (enGenome)	7	10	11	11	11	11	75 ± 10.5	0.49 ± 0.09	0.16	1.64 ± 1.69	3	4
3	Team 14 (TCS)	5	10	11	11	11	11	75 ± 10.6	0.34 ± 0.1	0.8	1.07 ± 1	3	11
2	Team 14 (TCS)	4	10	11	11	11	11	75 ± 11.1	0.34 ± 0.1	0.8	1.07 ± 1	3	11
1	Team 5 (Exomiser)	6	9	11	12	12	12	73.2 ± 8.8	0.47 ± 0.1	0.87	1.14 ± 1.29	6	6
3	Team 12 (Lichtarge)	8	10	10	10	11	11	72.1 ± 12.7	0.32 ± 0.1	0.95	1.71 ± 2.05	7	19
2	Team 12 (Lichtarge)	5	10	10	10	11	11	72.1 ± 11.9	0.26 ± 0.09	0.95	1.79 ± 1.85	7	25
3	Team 11 (enGenome)	4	9	11	11	11	12	71.8 ± 10.8	0.35 ± 0.09	0.12	1.43 ± 1.65	9	10
4	Team 11 (enGenome)	7	10	10	10	10	10	71.4 ± 10.8	0.48 ± 0.1	0.19	0.79 ± 0.89	10	5
2	Team 5 (Exomiser)	6	10	10	10	10	10	71.4 ± 12.3	0.53 ± 0.1	0.61	1.71 ± 1.82	10	3
2	Team 11 (enGenome)	7	9	10	10	10	10	67.9 ± 12	0.43 ± 0.1	0.1	1 ± 1.47	12	8
1	Team 14 (TCS)	6	9	10	10	10	10	67.9 ± 12.3	0.45 ± 0.11	0.9	0.57 ± 0.85	12	7
3	Team 5 (Exomiser)	4	9	10	10	10	10	67.9 ± 12	0.34 ± 0.09	0.88	1.07 ± 1.64	12	11
2	Team 10	2	9	9	10	11	11	66.8 ± 12	0.26 ± 0.08	0.98	1.71 ± 0.91	15	25
1	Team 10	1	6	8	11	11	11	55.4 ± 10.9	0.14 ± 0.06	0.99	5.07 ± 5.15	16	39
2	Team 1	4	7	7	8	10	10	53.2 ± 13.1	0.36 ± 0.09	0.07	1.36 ± 1.22	17	9
2	Team 4 (DITTO)	3	7	7	8	10	10	53.2 ± 13.4	0.32 ± 0.08	0.86	1.71 ± 2.02	17	19
4	Team 4 (DITTO)	3	7	7	8	10	10	53.2 ± 12.8	0.32 ± 0.08	0.86	1.64 ± 2.02	17	19
1	Team 4 (DITTO)	3	7	7	8	10	10	53.2 ± 11.7	0.33 ± 0.08	0.86	1.57 ± 1.74	17	17
5	Team 4 (DITTO)	3	7	7	8	10	10	53.2 ± 11.7	0.33 ± 0.09	0.86	1.57 ± 1.74	17	17
3	Team 4 (DITTO)	3	7	7	8	10	10	53.2 ± 12.1	0.34 ± 0.09	0.86	1.5 ± 1.74	17	11
6	Team 4 (DITTO)	3	7	7	8	10	10	53.2 ± 12.4	0.34 ± 0.09	0.86	1.5 ± 1.74	17	11
4	Team 5 (Exomiser)	2	6	8	9	10	10	52.5 ± 11.7	0.24 ± 0.1	0.96	1.43 ± 1.45	24	30
1	Team 1	4	7	7	7	7	9	50.7 ± 12.6	0.34 ± 0.1	0.76	1.07 ± 0.92	25	11
4	Team 1	4	7	7	7	7	8	50.4 ± 15.1	0.27 ± 0.11	0.99	0.57 ± 0.51	26	22
1	Team 2 (AIBI)	5	6	7	8	10	10	49.6 ± 12.8	0.22 ± 0.12	0.96	0.29 ± 0.61	27	31
2	Team 2 (AIBI)	5	6	7	8	10	10	49.6 ± 11.6	0.22 ± 0.11	0.96	0.29 ± 0.61	27	31
5	Team 2 (AIBI)	4	6	7	8	10	10	49.6 ± 12.3	0.25 ± 0.13	1	0.14 ± 0.36	27	28
4	Team 2 (AIBI)	5	6	7	8	9	10	49.3 ± 11.9	0.25 ± 0.12	0.88	0.14 ± 0.36	30	28
3	Team 1	3	5	7	9	10	10	47.1 ± 11.1	0.27 ± 0.11	1	0.57 ± 0.51	31	22
5	Team 1	3	5	7	9	10	10	47.1 ± 10.4	0.27 ± 0.11	1	0.57 ± 0.51	31	22
1	Team 13	4	6	7	7	7	7	46.4 ± 13.3	0.17 ± 0.09	0.59	1.5 ± 2.98	33	35
6	Team 2 (AIBI)	5	5	7	8	10	10	46.1 ± 11.4	0.22 ± 0.1	1	0.29 ± 0.83	34	31
2	Team 8 (BORG)	3	5	6	8	12	13	46.1 ± 10	0.26 ± 0.09	0.36	0.64 ± 1.6	34	25
3	Team 2 (AIBI)	5	5	7	8	9	9	45.4 ± 11.8	0.13 ± 0.12	0.98	0.07 ± 0.27	36	41
2	Team 15	6	6	6	6	6	6	42.9 ± 13.6	0.08 ± 0.03	0.85	9.5 ± 3.88	37	49
1	Team 15	6	6	6	6	6	6	42.9 ± 13.8	0.14 ± 0.09	0.83	5.14 ± 4.94	37	39
2	Team 13	3	5	6	7	7	7	41.1 ± 12.2	0.18 ± 0.08	0.73	0.57 ± 1.16	39	34
4	Team 8 (BORG)	0	3	5	7	10	13	35.4 ± 9	0.15 ± 0.06	0.46	0.86 ± 2.07	40	37
3	Team 8 (BORG)	1	3	5	6	10	11	33.6 ± 9.7	0.17 ± 0.1	0.55	0.64 ± 1.39	41	35
1	Team 16	1	4	5	5	5	5	32.1 ± 11.1	0.12 ± 0.08	0.51	0.21 ± 0.43	42	42
1	Team 8 (BORG)	2	3	3	5	8	11	28.2 ± 9.9	0.15 ± 0.08	0.38	2.86 ± 3.18	43	37
5	Team 5 (Exomiser)	0	3	4	4	5	7	26.4 ± 10.6	0.1 ± 0.06	0.98	2 ± 2.48	44	48
1	Team 7 (Uniss)	1	2	4	6	6	7	25.4 ± 9.6	0.11 ± 0.07	1	1.71 ± 1.73	45	47
3	Team 16	1	2	4	5	6	6	23.9 ± 9.4	0.12 ± 0.09	0.51	0.21 ± 0.43	46	42
2	Team 16	1	2	4	4	6	7	23.2 ± 9.9	0.12 ± 0.1	0.51	0.21 ± 0.43	47	42
2	Team 3 (Bologna Biocomputing Group)	1	2	3	5	6	6	22.1 ± 8.7	0.06 ± 0.04	1	5.64 ± 8.04	48	50
4	Team 16	1	2	2	2	2	2	14.3 ± 10	0.12 ± 0.08	0.51	0.21 ± 0.43	49	42
1	Team 3 (Bologna Biocomputing Group)	1	1	1	1	1	1	7.1 ± 6.5	0.12 ± 0.08	1	0.21 ± 0.43	50	42
1	Team 6	0	0	0	0	0	0	0 ± 0	NA	NA	NA	51	51
2	Team 6	0	0	0	0	0	0	0 ± 0	NA	NA	NA	52	52

For each team and model, the number of detected causal variants at rank 1, 1–5, 1–10, 1–20, 1–50, and 1–100 is displayed out of a maximum of 14. The mean rank points (maximum 100) and F-max value (maximum 1) assessment metrics are displayed with the bootstrapped SE (see Methods). The F-max producing EPCR values and the mean number of predictions per proband at or above this threshold are displayed with the SD. Model performance is ranked separately for each of the two metrics. SE, standard error; SD, standard deviation; F-max, maximum F-measure; EPCR, estimated probability of causal relationship

One of the top performing models from Team 9 (Invitae Moon) was able to prioritize 13 of the 14 causal variants within the top five rank positions, followed by Team 12 (Lichtarge) with 12, Team 11 (enGenome) and Team 14 (TCS) tied with 10, and Team 5 (Exomiser) with 9.

Following assessment of model performance, the assessor was unblinded to the identity and methods of the participating teams. The wide variability in methodology, spanning stepwise filtering approaches to machine learning and artificial intelligence, did not allow for a comprehensive analysis nor use of statistical tests. A qualitative review of the methods, summarized in Table 3, demonstrated decreased performance when one or more of the following features were not considered by the method: i) variant call quality; e.g., depth, genotype quality, and allele balance (resulting in the inclusion of sequence artifacts into submissions), ii) variant allele frequency; e.g., rare in large scale population databases such as gnomAD and TOPMed, iii) variant deleteriousness prediction; e.g., use of in silico tools and/or training on reported variants in clinical databases such as ClinVar and HGMD, iv) familial segregation within the provided dataset and inheritance mode of the respective gene, and v) relevance of the putative causal variant(s) to the proband’s phenotype. Some teams considered all of these features, yet the models did not identify many diagnostic variants, presumably due to the specific methodology used, information sources, and thresholds selected. In a small number of cases, the selected features excluded the causal variant, due to i) focusing on specific variant consequences (e.g., frameshift, nonsense, and/or missense), ii) not including compound heterozygous variants, iii) using hard thresholds for in silico deleteriousness prediction, iv) focusing on specific lists of disease-associated genes, and v) not considering sex-limited expression as part of segregation. For the remaining missed diagnoses, it is not possible to determine if the causal variant was excluded by the model or if it was prioritized below the 100-variant limit of the challenge. Detailed methods descriptions are provided for 11 of the 16 participating teams in the Additional file 1.

**Table 3:** Summary of method features

Model #	Team	Variant call quality	Variant allele frequency	Variant deleteriousness prediction	Familial segregation	Relevance to phenotype	Limited to human disease-associated genes	Limited to coding regions	Submitted compound heterozygous variants
1	Team 9 (Invitae Moon)	Yes	Yes—≤ 2% gnomAD, plus more common P/LP variants	Yes—trained on ClinVar and in-house classifications	Yes—plus incomplete penetrance	Yes	Yes—Apollo database	Yes—plus known P/LP non-coding variants	Yes
1	Team 12 (Lichtarge)	Yes	Yes	Yes—Evolutionary Action	Yes	Yes	Yes—HPO, DisGeNet, ClinVar, HumSavar, literature	Yes—frameshift, nonsense, and missense only (excluded causal variant in P19)	Yes
2–3	Team 12 (Lichtarge)	Yes	Yes	Yes—Evolutionary Action	Yes	Yes	Yes—VarElect NGS Phenotyper	Yes—frameshift, nonsense, and missense only (excluded causal variant in P19)	Yes
1–4	Team 11 (enGenome)	Yes	Yes	Yes	Yes	Yes	Yes—MedGen, Disease Ontology, Orphanet (excluded causal variants in P6 and P23)	No	Yes
1–3	Team 14 (TCS)	Yes	Yes	Yes (excluded causal variant in P16)	Yes (variant in P5 was predicted as compound heterozygous with another variant)	Yes	Yes—ClinVar, HPO, STRING, PubMed (excluded causal variant in P6)	Yes	Yes
1	Team 5 (Exomiser)	Yes	Yes—< 0.1% dominant, < 2% cmphet recessive 1000 Genomes, ExAC, gnomAD	Yes—REVEL, MVP (excluded causal variant in P24)	Yes—did not allow for sex-limited expression (excluded causal variant in P27)	Yes	No—also included model organisms and interacting proteins	Yes—plus known P/LP non-coding variants	Yes
2	Team 5 (Exomiser)	Yes	Yes—< 0.1% dominant, < 2% cmphet recessive 1000 Genomes, ExAC, gnomAD	Yes—REVEL, MVP (excluded causal variant in P24)	Yes—did not allow for sex-limited expression (excluded causal variant in P27)	Yes	Yes—OMIM, Orphanet (excluded causal variants in P6 and P23)	Yes—plus known P/LP non-coding variants	Yes
3	Team 5 (Exomiser)	No	Yes—< 0.1% dominant, < 2% cmphet recessive 1000 Genomes, ExAC, gnomAD	Yes—REVEL, MVP (excluded causal variant in P24)	Yes—did not allow for sex-limited expression (excluded causal variant in P27)	Yes	Yes—OMIM, Orphanet (excluded causal variants in P6 and P23)	Yes—plus known P/LP non-coding variants	Yes
4	Team 5 (Exomiser)	Yes	Yes—< 0.1% dominant, < 2% cmphet recessive 1000 Genomes, ExAC, gnomAD	Yes—REVEL, MVP (excluded causal variant in P24)	Yes—plus incomplete penetrance	Yes	Yes—OMIM, Orphanet (excluded causal variants in P6 and P23)	Yes—plus known P/LP non-coding variants	Yes
5	Team 5 (Exomiser)	No	Yes—< 0.1% dominant, < 2% cmphet recessive 1000 Genomes, ExAC, gnomAD	Yes—REVEL, MVP (excluded causal variant in P24)	Yes—did not allow for sex-limited expression (excluded causal variant in P27)	Yes	Yes—OMIM, Orphanet (excluded causal variants in P6 and P23)	No	Yes
1–6	Team 4 (DITTO)	Yes	No	Yes—trained on ClinVar and HGMD classifications	No	Yes—Exomiser	No	No	No (excluded causal variant in P5)
1–6	Team 2 (AIBI)	No	Yes—< 5%	Yes—REVEL	Yes	Yes—Phenolyzer	Yes—HPO	Yes	Yes
1	Team 13	Yes	Yes—< 0.1%	Yes—MutPred2 (excluded causal variant in P27)	Yes—absent in parent if data avaliable (excluded causal variants in P5 and P27)	Yes—HPO including interacting proteins	No	Yes—missense only (excluded causal variants in P5, P7, P16, P19, and P22)	No (excluded causal variant in P5)
2	Team 13	Yes	Yes—< 0.1%	Yes—REVEL (excluded causal variant in P27)	Yes—absent in parent if data available (excluded causal variants in P5 and P27)	Yes—HPO including interacting proteins	No	Yes—missense only (excluded causal variants in P5, P7, P16, P19, and P22)	No (excluded causal variant in P5)
1–4	Team 8 (BORG)	Yes	Yes—≤ 1% 1000 Genomes, ExAC, gnomAD	Yes—CADD	Yes	Yes—HPO	-	-	No (excluded causal variant in P5)
1	Team 7 (Uniss)	–	Yes—≤ 1% gnomAD	Yes	-	Yes—HPO, Orphanet	Yes	-	No (excluded causal variant in P5)
1	Team 3 (Bologna Biocomputing Group)	–	Yes—≤ 1% gnomAD	-	Yes	Yes—eDGAR, PhenPath (excluded causal variants in P4, P7, and P21)	Yes—eDGAR, PhenPath	Yes—frameshift, nonsense, and missense only (excluded causal variant in P19)	No (excluded causal variant in P5)
2	Team 3 (Bologna Biocomputing Group)	–	Yes—≤ 1% gnomAD	-	Yes—de novo and homozygous only (excluded causal variants in P2, P5, P6, P11, P22, and P27)	Yes—eDGAR, PhenPath (excluded causal variants in P4, P7, and P21)	No	Yes—frameshift, nonsense, and missense only (excluded causal variant in P19)	No (excluded causal variant in P5)

The 11 teams providing methods details are displayed with key method features and, where possible to determine, the explanation for excluded causal variants

### Variant detection by top performing teams

**Team 9 (Invitae Moon):** The Invitae Moon team submitted one model and predicted the causal variant(s) in 13 of 14 solved families within the top five ranked variants, nine at rank position one. At the F-max producing EPCR threshold, a mean of one variant was prioritized per proband (14 in total, 9 causal). The model’s performance ranked first by the mean rank points metric and second by F-max. Only one diagnosis was missed, a de novo variant in *BICC1* for P21, presenting with unilateral multicystic kidney dysplasia and severe infantile onset neutropenia.

Moon™ (Invitae, San Francisco, CA) is an automated analysis software package developed to prioritize likely causative variants from genome or exome sequencing data. Variant prioritization is achieved by an algorithm incorporating i) the patient’s clinical and sequencing data, ii) parental sequencing data and affected status, iii) curated gene-phenotype associations, and iv) variant annotations, including gnomAD frequency, variant effect predictions, ClinVar submissions and Invitae classifications (internal data). Gene-phenotype associations are maintained in the “Apollo” database by trained genetic scientists at Invitae, and kept up-to-date by daily scanning of the published medical literature for new gene-phenotype associations, followed by manual review and curation of relevant information; HPO terms, the number of patient observations for each HPO, range of disease onset for reported individuals, and the reported inheritance pattern and pathogenic mechanism for the gene. Variants were submitted only for genes that have already been associated with Mendelian disorders in scientific literature. Moon™ is a commercial product available for paid licensed use and was used in an automated fashion.

**Team 12 (Lichtarge):** The Lichtarge team at the Baylor College of Medicine submitted three models. Their top performing model by both metrics, model 1, predicted the causal variant(s) in 12 of 14 solved families within the top five ranked variants, of which nine were at rank position one. At the F-max producing EPCR threshold, a mean of 1.21 variants were prioritized per proband (17 total, 10 causal). The model’s performance ranked second by the mean rank points metric and first by the F-max metric. The model did not identify the causal variant(s) for two probands (P6 and P19).

The Lichtarge team developed scoring systems to prioritize missense, nonsense, and frameshift variants. The team left silent, splicing, and non-coding variants out of their analysis, such as the causal variant of P19. They used the Evolutionary Action method [33] to predict the functional consequences of the missense variants, and accounted for variant call quality, population allele frequency, variant segregation pattern in the families (de novo, X-linked dominant males, and autosomal recessive), the ability of each gene to tolerate mutations (unpublished score based on Evolutionary Action), and known gene associations with the patient’s phenotype. Their top performing model, model 1, prioritized the variants according to the predicted probability for loss of gene function, in contrast to models 2 and 3 that prioritized variants above a threshold for predicted loss of gene function, according to their association to the provided phenotypes. Merging of the variants prioritized for different inheritance modes was performed manually using the predictor’s judgment to provide a single submission. These tools are in-house, involve automated and manual analysis, and are not publicly available at this time; more information can be obtained by contacting the authors.

**Team 11 (enGenome):** The enGenome team submitted four models. Their top performing model by both metrics, model 1, predicted the causal variant(s) in 10 of 14 solved families within the top five ranked variants, of which seven were at rank position one, and predicted 11 of 14 overall. At the F-max producing EPCR threshold, a mean of 1.64 variants were prioritized per proband (23 total, 9 causal). The model did not identify the causal variant(s) for three probands (P6, P21, and P23) in their submission. However, with model 3, the enGenome team identified 12 causative variants of 14 overall.

The enGenome team applied ensemble and linear machine learning classifiers trained on the challenge training set. The features set used to identify the causative variant(s) relies on ACMG/AMP variant pathogenicity, computed through enGenome proprietary variant interpretation software eVai, [34, 35], as well as variant quality, family segregation and phenotypic similarity. ACMG/AMP classification is computed only if the gene is associated with at least one condition in databases such as MedGen (https://www.ncbi.nlm.nih.gov/medgen/), Disease Ontology (https://disease-ontology.org/), and Orphanet (https://www.orpha.net/) and phenotypic similarity metrics are computed only when the gene is known to be associated with at least one phenotype. This explains the diagnoses missed by enGenome in the test set (P6 and P23), as both causative genes (*KCND2* and *GNAI1*) were not associated with conditions in these databases when the models were trained. In one additional case (P21), the causative gene was not associated with phenotypes in these databases at the time of the challenge and was identified only by model 3. enGenome’s eVai platform is a commercial product available for paid licensed use and was used in an automated fashion.

**Team 14 (TCS):** The TCS team submitted three models. Their top performing models by mean rank points, models 2 and 3, predicted the causal variant(s) in 10 of 14 solved families within the top five ranked variants, with a maximum of five at rank position one, and predicted 11 overall. Collectively, the models did not identify the causal variant(s) for three probands (P6, P16, and P24). Their top performing model by F-max value was model 1, prioritizing a mean of 0.6 variants per proband at the F-max producing EPCR threshold (8 total, 5 causal).

The TCS team used a combination of in-house tools, “VPR” for variant prioritization and “PRIORI-T” [36] and “GPrio” for gene prioritization. Briefly, variants were ranked based on minor allele frequency, evolutionary conservation, in silico predictions of deleteriousness, and prior disease associations. PRIORI-T queries a rare disease heterogeneous association network with the HPO terms for each proband and outputs a ranked list of genes. GPrio calculates gene scores by two methods. The first is based on HPO-gene correlations reported in the HPO database (https://hpo.jax.org/app/) [15]. The second uses the STRING-DB database (https://string-db.org/) [37] to explore indirect hits through interacting genes with relevant HPO correlations. Based on different combinations of the tools, three prediction models were submitted, described in the Additional file 1. The TCS tools are in-house, involve manual analysis, and are not publicly available at this time; more information can be obtained by contacting the authors.

**Team 5 (Exomiser):** The Exomiser team submitted five models. Their top performing model by mean rank points, model 1, predicted the causal variant(s) in nine of 14 solved families within the top five ranked variants, of which six were at rank position one, and predicted 12 overall. The model did not identify the causal variant(s) for two probands (P24 and P27) in their submission. Their top performing model by F-max value was model 2, prioritizing a mean of 1.71 variants per proband at the F-max producing EPCR threshold (24 total across all 14 probands, 10 causal).

The open-source Exomiser tool (version 13.0.0) [38] was run using the latest databases (2109) at time of analysis (Sep 2021), along with a local frequency file generated from 86 non-training samples where AC > 1. A maximum of 100 variants per model were returned for all candidates with an Exomiser score > 0.2 based on Exomiser’s ranking with no further manual intervention. Model 1 used the recommended default Exomiser settings where high quality (FILTER = PASS in input VCF), rare, segregating, coding variants were prioritized based on minor allele frequency, predicted pathogenicity and the similarity of the patient phenotypes to reference genotype to phenotype knowledge from human disease and model organism databases along with neighbors from the STRING-DB protein–protein association databases (https://string-db.org/) [37]. Model 2 used the same settings except only reference human disease knowledge was used. Model 3 extended the model 2 analysis to all variants in the VCF, rather than just the high-quality ones. Model 4 extended the model 2 analysis to allow incomplete penetrance where the prioritized variants can also be present in unaffected family members. Model 5 extended the model 3 analysis to non-coding variants in the genome sequence using the Genomiser variant of Exomiser [39]. The two diagnoses missed by model 1 were due to a sex-limited phenotype in one case and a low predicted pathogenicity by REVEL and MVP [10, 40] in the other. In the latter case, this variant has now been deposited in ClinVar and would be a top-ranked candidate if rerun due to the ClinVar whitelisting feature of Exomiser. For the three diagnoses ranked outside the top five, two involved disease-gene associations that were in the published literature but not present in OMIM at the time of analysis; these would be highlighted as top-ranking candidates if rerun now (May, 2023). Exomiser is open source and freely available and was used in an automated fashion.

### Reanalysis of solved families

Given the high performance of these models, we reanalyzed the solved families in which models ranked variants higher than the causal variants identified by the RGP team in the answer key, to determine if they may contribute to disease or represent a more likely causal diagnosis; however, no compelling variants were found. To illustrate this, a detailed review of the variants prioritized by one of the top performing teams, Team 9 (Invitae Moon) in four probands (P2, P6, P7, and P11) is provided in the Additional file 1.

### Review of “difficult to predict” diagnoses

In genomics-driven diagnostics, failure to recognize causal variants and to falsely prioritize non-causal variants are recognized complications [5, 41]. We therefore reanalyzed families in the answer key for which predictors consistently failed to prioritize the causal variant(s). Several of these are described below.

The most poorly predicted diagnosis was *KCND2* (c.1207C > G, p.Pro403Ala, ENST00000331113) in P6, a patient presenting with infantile-onset bilateral sensorineural hearing impairment, blindness, retinal dystrophy, hypotonia, chorea, profound global developmental delay, intellectual disability, and dystonia. Across all models, the causal variant was never reported at rank position one, was ranked at position 2–5 by just three models, and was only listed by 26 of 52 models (50%) across all variant predictions. This heterozygous ACMG/AMP LP missense variant in *KCND2* explains the patient’s phenotype [42], is predicted to be deleterious by in silico prediction (REVEL 0.84—PP3 Moderate) [8, 10], and is absent from large population databases (gnomAD and TOPMed) [3, 12]. However, only duo sequencing was available for this family, from the proband and unaffected father; therefore, the de novo status of the variant remains unconfirmed. This hinders models in prioritizing the variant. Calculating the mean rank points metric separately for families with proband-only or duo data versus those with trio or quad data, demonstrates a significant improvement in model performance with trio or quad data (paired Student's T-Test p-value 0.00086) (Fig. 2C). *KCND2* is also not yet reported in the OMIM database as Mendelian-disease associated (last accessed April 2023). Models limiting their assessment to reported Mendelian-disease associated genes, may fail to prioritize this causal variant (Table 3), highlighting the importance of OMIM and similar databases to the medical genomics community and the need to be able to represent novel gene-disease associations more rapidly. One such option for laboratories reporting novel Mendelian gene-disease relationships is to deposit them in the Gene Curation Coalition (GenCC) Database (https://thegencc.org/) allowing more rapid dissemination of findings to the community as well as the aggregation of many public and private gene-disease databases [43].

The second most poorly predicted diagnosis was *PI4KA* in P5, a patient presenting with global developmental delay, poor coordination, hypotonia, and spasticity, with an MRI-brain demonstrating cerebral hypomyelination and a dysplastic corpus callosum. Across all models, the two causal variants in this recessive gene were found at position 1–5 in nine models and were only listed by 18 of 52 models (35%) across all submitted variants. The first variant is a P nonsense variant (c.1852C > T, p.Arg618Ter, ENST00000255882; ACMG/AMP criteria applied: PVS1, PM2, PP1, PP3, and PP4). The second is a LP missense variant (c.4990G > A, p.Asp1664Asn, ENST00000255882; ACMG/AMP criteria applied: PP1, PP3, PP4, PM1 Supporting, PM2, PM3). Plausible explanations for the low prediction rate include: i) the requirement for models to jointly prioritize compound heterozygous variants, and ii) the need to consider phenotype expansion, as at the time of the challenge PI4KA had only be associated with polymicrogyria, cerebellar hypoplasia, and arthrogryposis [44]. Not all teams included compound heterozygous variants in their submissions (Table 3) despite several cases of recessive inheritance being included in the training set. As with the *KCND2* family, this family was also sequenced as a duo (proband and unaffected father). The nonsense variant is paternally inherited, requiring the assumption that the missense variant is maternally inherited or de novo on the maternal haplotype, to constitute a recessive diagnosis.

The third most poorly predicted diagnosis was a splice acceptor variant in *CLTC* (c.1534del, p.Val512LeufsTer11, ENST00000621829), a gene associated with intellectual disability in OMIM (MIM: 617,854). The proband (P19) presented with global developmental delay, hearing impairment, severely reduced visual acuity, constipation, hyperbilirubinemia, pulmonary arterial hypertension, and intracranial hemorrhage. This variant was ranked at position 1–5 by 13 models and was only listed by 18 of 52 models (35%) across all submitted variants. This de novo heterozygous LP splice acceptor variant (ACMG/AMP criteria applied: PS2, PM2, PVS1 Moderate) is predicted to cause a frameshift leading to a premature stop codon 11 amino acids downstream (in exon 10 of 31) in a highly loss-of-function constrained gene and is absent from large population databases. Since the challenge, the *CLTC* variant has been reported as LP in ClinVar by an independent submitter in association with intellectual disability (ClinVar variation ID: 811,442). This variant arises at an acceptor splice site in the gene, thereby outside of the protein-coding region defined by some models (Table 3).

Finally, the fourth most poorly predicted diagnosis was *TUBB8* in P27 (c.1039A > G, p.Asn347Asp and c.1033C > T, p.Leu345Phe, ENST00000568584), a female proband sequenced as a quad with her affected female sibling and both unaffected parents. In this family, two causal variants in *TUBB8* were identified, inherited in *cis* from the unaffected father. Carriage of the causal variants by the unaffected father is explained by sex-limited expression of the oocyte maturation defect disease phenotype in females (MIM: 616,780). To prevent exclusion of these variants, the model would need to take sex-limited expression into consideration. This was achieved by some models by allowing for incomplete penetrance (Table 3).

### Summary of variant predictions in unsolved probands

Through reanalysis of the 16 unsolved families, directed by the submitted variant predictions from the top 10 teams, two additional families (12.5%) received a genetic diagnosis. The first, by the detection of a de novo splice region variant in *TCF4* (c.1228 + 3G > T, ENST00000398339), prioritized by eight models in total, submitted by Team 9 (Invitae Moon, model 1 at rank 1), Team 5 (Exomiser, model 1–2 at rank 1 and model 3 at rank 2), and Team 11 (enGenome, model 2 and 4 at rank 1, and model 1 and 3 at rank 2). The second, by the detection of compound heterozygous frameshift (c.706del, p.Arg236GlyfsTer8, ENST00000175506) and deep intronic (c.1137 + 200_1137 + 205del, ENST00000175506) variants in *ASNS*, submitted as a biallelic prediction by Team 11 (enGenome, model 1, 2, and 4 at rank 1, and model 3 at rank 2) only. Notably, four additional models from Team 9 (Invitae Moon, model 1, rank 7) and Team 2 (AIBI, model 1, 5, and 6 at rank 83–91) prioritized the *ASNS* frameshift variant only. In both probands, the variant(s) impact on the transcript were functionally validated by RNA sequencing and were returned to the families following confirmation in a CLIA certified laboratory (Additional file 2: Table S3).

In a further six unsolved families, variants in putative novel disease genes were prioritized (Additional file 2: Table S3). For four of the six, a submission had already been made by the RGP team to Matchmaker Exchange (*TPPP* in P9, *KCNH8* in P14, *KLHL13* in P15, and *THAP12* in P18). For the remaining two, new submissions were made (*MRPL54* in P25 and *FRY* in P26). To date, Matchmaker Exchange matches warranting further consideration of these candidate genes have not been received, however, functional studies are underway for some candidates through the GREGoR consortium (https://gregorconsortium.org/). Across the remaining unsolved families, no variants identified were deemed of comparably high interest by the RGP team to pursue by functional studies or submission to Matchmaker Exchange.

**Fig. 3:**
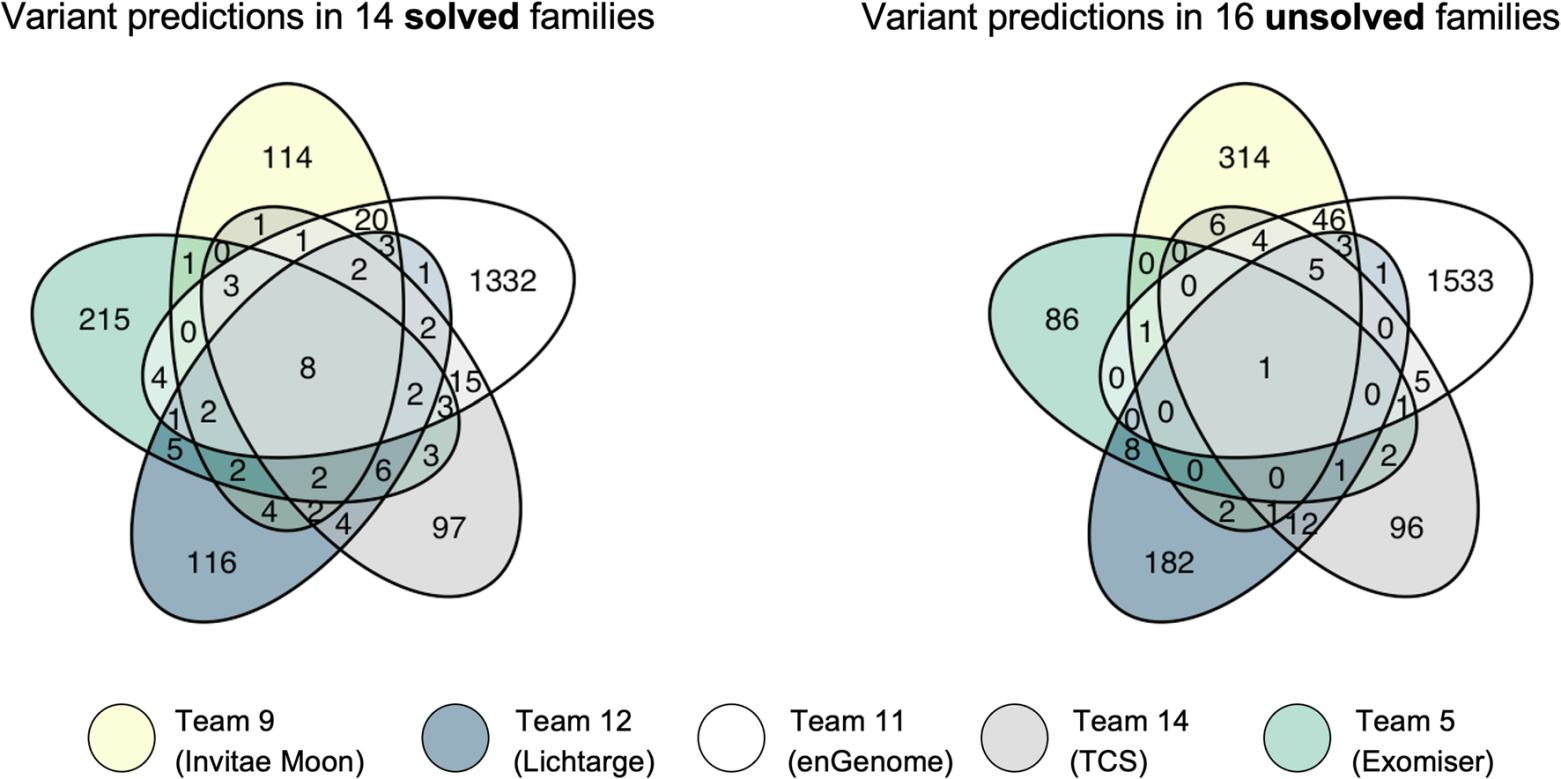
Concordance in the variant predictions submitted by top five performing teams in the solved and unsolved families. Venn diagrams demonstrating the overlap in the variant predictions submitted across all probands in the solved families (left) compared to the unsolved families (right) between top performing teams

Overall, there was more limited concordance in the variant predictions submitted between the top performing models in the unsolved families, compared to the solved families (Fig. 3); and the vast majority of prioritized variants in the unsolved families did not merit further evaluation after review.

The variants that did not merit further review in the unsolved families mostly fell into one or more of the following categories: i) heterozygous variants in dominant disease genes (according to the reported mode of inheritance in OMIM) inherited from an unaffected biological parent, and where incomplete penetrance is not expected based on current understanding, ii) heterozygous variants in dominant disease genes present in large population databases at an allele frequency higher than consistent with the prevalence of disease, where incomplete penetrance is not expected, and iii) single heterozygous variants in recessive disease genes that are unable to constitute a diagnosis without a compound heterozygous variant. For families with a single recessive variant and at least partial phenotype overlap with the reported phenotype, an SV call set generated by the GATK-SV pipeline was analyzed and the gene was manually reviewed in IGV with the aim to identify an SV in *trans*. This analysis did not result in the detection of any additional variants of interest.

To provide one example, a heterozygous maternally inherited missense variant in *GRIK2* was prioritized at rank position one by Team 9 (Invitae Moon) in P15. The variant (c.1066G > A, p.Gly356Arg, ENST00000421544) is predicted to be deleterious by in silico predictions (REVEL 0.95—PP3 Strong) [45] and is absent in large population databases. *GRIK2* is associated with dominant neurodevelopmental delay, impaired language, and ataxia (MIM: 619580) and with recessive intellectual disability (MIM: 611092). The dominant form of disease results from de novo gain-of-function variants clustering in a specific domain of ionotropic glutamate receptors, proven to affect channel kinetics and function [46, 47]. As the *GRIK2* variant prioritized by Team 9 is inherited from the unaffected mother and falls far outside of this functional domain, it is inconsistent with being the cause of dominant disease under the assumption of complete penetrance, whereby every individual who has the variant shows signs and symptoms of the disease. The recessive form of disease results from biallelic loss-of-function variants [48, 49]. As the proband is lacking a second biallelic variant, the variant can also be deprioritized as a cause of recessive disease.

### Returnable diagnoses identified in two unsolved families

**Fig. 4:**
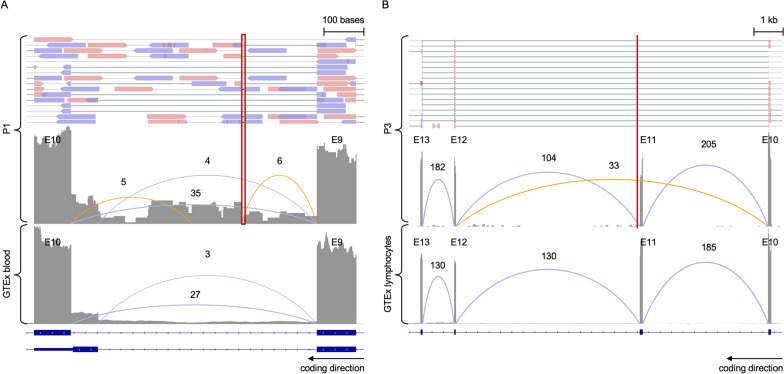
Confirmatory RNA sequencing in P1 and P3. For both **A** and **B**, in the top panel, paired end reads from the RNA sequencing BAM file are displayed for the proband. In the lower panels, the RNA sequencing read pileup tract is displayed with the novel (orange) and known (blue) junctions annotated in the proband and in aggregated data from GTEx controls, respectively. Beneath, the gene transcript isoforms are displayed. **A**, RNA sequencing analysis performed on blood in P1 compared to normalized GTEx blood samples (n = 755) (21). The results for *ASNS* (displaying exon 9 and 10) demonstrate evidence of splice disruption due to a deep intronic indel (indicated by the red box in the proband) with cryptic exon creation and intron 9 read-through. **B**, RNA sequencing analysis performed on an EBV-transformed lymphoblastoid cell line (LCL) in P3 compared to normalized GTEx lymphocyte samples (n = 174). The results for *TCF4* (displaying exon 10 to 13) demonstrate evidence of splice disruption due to a near-splice variant (indicated by the red line in the proband) with skipping of exon 11 in approximately 20% of reads. E, exon

For P1, Team 11 (enGenome) prioritized compound heterozygous putative loss-of-function variants in *ASNS* at rank position 1–3 across four submitted models; a maternally inherited frameshift variant (c.706del, p.Arg236GlyfsTer8, ENST00000175506) and a paternally inherited deep intronic 6 base pair deletion (c.1137 + 200_1137 + 205del, ENST00000175506). *ASNS* is a disease gene associated with asparagine synthetase deficiency (MIM: 615,574) and is a phenotype match for the proband, who presented with Lennox-Gastaut syndrome, infantile spasms, microcephaly, hypotonia, nystagmus, optic nerve hypoplasia, partial agenesis of the corpus callosum, and delayed myelination. Loss-of-function of *ASNS* is an established disease mechanism in autosomal recessive asparagine synthetase deficiency [50, 51]. The frameshift variant is rare in large population databases (absent in gnomAD, reported in 1/264,690 alleles in TOPMed) and has recently (Feb, 2022) been reported as P in ClinVar (ClinVar variation ID: 1411238). The variant leads to a premature stop codon in the middle of the gene, in exon six of 13, and is expected to result in a truncated protein. The variant is classified as LP according to ACMG/AMP guidelines (criteria applied: PVS1 and PM2 Supporting). The deep intronic indel between exons 10 and 11 (200 bp away from the exon) is absent from large population databases and has a moderate SpliceAI score (0.2) [11] predicting acceptor gain. RNA sequencing analysis performed on blood from the proband demonstrated evidence of complex splice disruption, including intron retention and novel exon creation, leading to a premature stop codon in the middle of the gene (Fig. 4A). In light of this evidence, the variant was classified as LP according to ACMG/AMP guidelines (criteria applied: PS3, PM3, and PM2 Supporting). *ASNS* was deemed a clinical fit by the family’s local physician. A cerebrospinal fluid (CSF) asparagine level was measured in the proband at 7-months of age and was found to be within normal range. Though low CSF asparagine level would further support the diagnosis, normal levels have previously been reported in patients with *ASNS* defects, due to limitations in the sensitivity of the assay [50, 52]. The family is now pursuing oral asparagine therapy.

In P3, three top performing teams, Team 9 (Invitae Moon), Team 5 (Exomiser), and Team 11 (enGenome), prioritized a de novo variant in *TCF4* (c.1228 + 3G > T, ENST00000398339), a disease gene associated with dominant Pitt-Hopkins syndrome (PHS, MIM 610954). This splice region variant has a moderate SpliceAI score (0.72) predicting donor loss and is absent from large population databases. Moreover, it is a putative loss-of-function variant in a highly loss-of-function constrained gene (pLI score 1, LOEUF 0.22, gnomAD) for which loss-of-function is an established disease mechanism [53]. This *TCF4* variant was flagged during analysis in *seqr* by the RGP team. However, at the time, it was considered non-compelling due to the absence of classical PHS features in the proband, such as dysmorphism, including a large beaked nose, wide mouth, fleshy lips, and clubbed fingertips, and abnormal breathing patterns, presenting as hyperventilation episodes. The phenotypic spectrum of *TCF4* has, however, since been expanded to include neurodevelopmental delay in the absence of classical PHS [54]. Moreover, upon re-contacting the family for additional clinical information and to request photographs, abnormal breathing patterns and mild dysmorphic features supporting PHS were confirmed. The variant has recently (Aug, 2021) been independently reported in ClinVar as LP (variation ID: 1,204,043), and has been reported in a study generating patient-specific induced pluripotent stem cells to model PHS [55]. RNA sequencing analysis performed on cultured lymphoblasts from the proband demonstrated evidence of splice disruption with exon skipping in the middle of the gene, in exon 11 of 20 (Fig. 4B). The variant was thereby classified as LP according to ACMG/AMP guidelines (criteria applied: PVS1 and PM2 Supporting).

## Discussion

The CAGI6-RGP challenge was designed to assess the state of the art in genome interpretation for rare diseases in a real-life clinical diagnostic setting. For this reason, rather than selecting families with readily detectable variants that had previously been reported in patients and deposited in ClinVar, we specifically selected families that had unreported variants that are often classified as VUS by clinical laboratories without careful consideration (which were often missense variants), and included a predominance of families for which no causal variant was yet identified following current field standards.

We selected two numeric assessment metrics and found wide variability in the performance of models to detect the causal variants. This variability was expected for a challenge encouraging participation from teams experimenting with novel models side-by-side to those with well-established models and infrastructure. The first assessment metric was the mean rank points metric, a simple weighted point allocation metric developed to reward models ranking causal variants as highly as possible, with the number of awarded points falling rapidly as variants dropped in the ranking. We selected this metric as variant curation following ACMG/AMP guidelines [6–8] requires considerable time and is likely to be undertaken for only a handful of highly ranked variants in the clinical setting. This metric did not take into consideration the team’s EPCR values, which were assessed by our second metric, the F-max value. High performance according to the F-max value required models to have a consistent scoring system across all probands, and rewarded models able to stratify causal from non-causal variants at an optimized F-max producing EPCR threshold. A reliable threshold for causal variant detection supports an analysts’ decision to conclude analysis of a diagnostic genome and deem the result inconclusive, as opposed to arbitrarily curating the top 5 or 10 ranked variants. There were minor discrepancies between the performance of the models depending on the assessment metric used; however, the top performing teams were reasonably consistent. No single model ranked the causal variant highest across all probands, indicating different strengths in different scenarios. A qualitative review of the methods was able to determine the key model features (call quality, allele frequency, predicted deleteriousness, segregation, and relevance to phenotype) along with the reason for the exclusion of specific causal variants by some models (e.g., due to not considering non-coding variants or genes without a reported disease association). It was not possible, however, to conclude exactly why some models ranked a variant highly and some not. Most of the top performing models were able to prioritize the more challenging diagnoses, such as compound heterozygous variants in a family with incomplete data for phasing that required openness to phenotype expansion (*PI4KA*), and an inherited dominant variant from an unaffected parent with sex-limited expression (*TUBB8*). Three of the top performing teams, Team 11 (enGenome), Team 9 (Invitae Moon), and Team 5 (Exomiser), also contributed to the diagnosis of previously unsolved probands. Both of these diagnoses involved non-coding variants and were returned to the families following functional validation by RNA sequencing. This included compound heterozygous frameshift and deep non-coding variants in *ASNS* prioritized by Team 11 (enGenome) that revealed a targeted therapy of potential clinical benefit, oral asparagine therapy [56], and a de novo near-splice variant in *TCF4*.

Looking into the variant predictions in families remaining unsolved, we found that many prioritized variants did not segregate in the family, had a higher allele frequency than feasible for the disease, were inconsistent with reported mode of inheritance, had no clear functional consequence based on current knowledge and in silico deleteriousness prediction tools, or had limited consistency with the patient's phenotype to be considered plausible, despite most models taking these features into consideration. This raises a number of issues. First, our reanalysis of the unsolved families assumed monogenic cause and complete penetrance (unless incomplete penetrance was previously reported for the gene), and we deprioritized inherited variants from unaffected parents and variants with higher-than-expected allele frequencies that may, arguably, play a role in incompletely penetrant or higher-order oligogenic disease. Second, beyond cases of a clear phenotype consistency, such as the newly diagnosed *ASNS* proband, we did not consider non-coding variants to be high priority for functional follow-up without in silico prediction of a splicing alteration. The strength of models recognizing deleterious non-coding variants may therefore be limited by the design of this challenge and current knowledge, and would be better positioned to perform well in a CAGI challenge with a functional readout of variant consequence as the answer key. Functional interpretation of variants in both known and novel disease genes is an ongoing challenge in rare disease diagnostics, eased by integration of high-throughput functional “omics” data like RNA sequencing and quantitative proteomics, and multiplex assays of variant effect (MAVE) [57] including deep mutational scanning, massively parallel reporter assays, and saturation genome editing [58–60]. Third, there was limited phenotype consistency, indicating room for improvement in phenotype matching methodology. For each of these scenarios, it is reasonable to consider that some of the variants identified by models in the challenge may in the future be reclassified as P/LP as evidence accumulates.

The CAGI6-RGP challenge has several limitations: i) Unlike other CAGI prediction challenges where teams are tasked to predict functional consequences for variants where the enzyme activity had been quantitatively measured, there was no definitive answer key for this challenge. The answer key used in assessment reflects the best of our team’s abilities to identify causal variants applying available evidence and following current clinical field standards. ii) We proactively selected families where the causal variant was not reported as disease-causing in ClinVar or HGMD at the time of challenge design, in order to task the models to identify novel causal variants, and delayed submission of the variant to ClinVar for the duration of the challenge. This skewed the spectrum of selected families toward novel heterozygous de novo variants and resulted in the inclusion of only one compound heterozygous recessive diagnosis. iii) The challenge was limited to SNVs and small indels, and did not include other classes of variant; e.g., SVs, tandem repeat expansions, mitochondrial DNA variants, or epigenetic alterations. iv) The answer key was limited to genes associated with disease in the literature and did not include novel candidate genes as it is more difficult to assert an answer as correct in the case of a proposed gene discovery. v) Neither assessment methodology precisely models the clinical challenge of balancing sensitivity (for discovery) with specificity (for clinical reporting), which are two very different goals. Moreover, predictors were not informed of the specific assessment metrics and how this might impact the perceived performance of their model, as this was developed at the time of assessment rather than challenge design. For the scale of the data and with subsequent analysis, however, our selected assessment metrics effectively identified the strength and weakness of different prediction models. vi) With the exception of top performers, teams were not required to be identified or to submit detailed methods. We appreciate that many teams were willing to be identified and provide this information. vii) We did not stipulate that entries could not undergo manual curation prior to submission and cannot mitigate the risk of model performance reflecting, to some extent, the result of human review. viii) The influence of proprietary databases on model performance could not be quantified. The large volume of unpublished sequencing and phenotype data mined to curate variants and for model development by teams with diagnostic laboratories may have given an advantage (for example, key considerations enabling the upgrade of a variant from VUS to P/LP include report of a specific clinical phenotype and identification of a variant in multiple unrelated individuals). ix) Early participation in RGP was predominantly by families of European descent, reflected in the case selection for the CAGI6-RGP challenge. We hope to have a more US-representative cohort in future challenges and have been working on approaches to diversify participation [30]. To improve future clinical diagnostic challenges, we recommend including a wider array of variant types and modes of inheritance, requiring teams to submit the automated output of the model without human review/reprioritization, requesting estimates of run time and cost to gain an appreciation for the computational power required and burden of the model, and appealing to teams with proprietary databases to submit a second entry limited to publicly available data only.

Overall, CAGI challenges provide essential information about methods in the field, evaluating both commercial and non-commercial tool performance on unpublished datasets through independent assessment. The CAGI6-RGP challenge has seen among the highest participation of teams to date, in particular increased uptake from industry, even with the higher bar to participate by requiring predictors to sign a data use agreement. The challenges are, however, only as good as the amount of participation from academic and industry teams, as well as clinical diagnostic laboratories, and involvement is greatly encouraged and appreciated.

## Conclusions

Computational models for genome analysis were found to be highly variable in terms of methodology and performance for rare disease diagnosis. Models weighing call quality, allele frequency, and predicted deleteriousness, in the context of segregation and phenotype, were effective in identifying causal variants, especially when variants could be phased with parental sequencing. Models open to phenotype expansion and non-coding variants were able to capture more difficult diagnoses, and could do so without hindering the ability to highly rank a small number of candidates for review. Overall, we find that computational models significantly aid genome interpretation and can act as clinical decision support tools. Their output does, however, require detailed review and conservative assessment of prioritized variants against established criteria for use in diagnostics.

### Supplementary Information

**Additional file 1.** Supplementary Information (Methodology of participating teams, Reanalysis of highly ranked variants by top performing teams in solved families, Supplemental Figures 1–2, and Supplemental References).

**Additional file 2.** Supplementary Tables 1–3.

## Data Availability

Sequence CRAM files and metadata for the Rare Genomes Project is available through the Broad Institute Data Use Oversight System (DUOS) at duos.broadinstitute.org under dataset IDs DUOS-000008 and DUOS-000143 or as part of the GREGoR consortium dataset via dbGaP study phs003047. Contact the authors for the sample ID code used in the CAGI challenge.

## Abbreviations

ACMG/AMP: American College of Medical Genetics and Genomics and the Association for Molecular Pathology
AD: Autosomal dominant
AFR: African/African American
AMR: Admixed American
AR: Autosomal recessive
ASJ: Ashkenazi Jewish
CAGI: Critical Assessment of Genome Interpretation
CSF: Cerebrospinal fluid
DM: Disease mutation
EPCR: Estimated probability of causal relationship
F-max: Maximum F-measure
GS: Genome sequencing
HPO: Human Phenotype Ontology
IGV: Integrative Genome Viewer
indel: Small insertion/deletion
LP: Likely pathogenic
NFE: Non-Finnish European
P: Pathogenic
PHS: Pitt-Hopkins syndrome
RGP: Rare Genomes Project
SAS: South Asian
SE: Standard error
SNV: Single nucleotide variant
SV: Structural variant
VCF: Variant call format
VEP: Variant Effect Predictor
VUS: Variant of uncertain significance
XLR: X-linked recessive
